# Ultrafast All‐Optical Switching and Active Sub‐Cycle Waveform Control via Time‐Variant Photodoping of Terahertz Metasurfaces

**DOI:** 10.1002/advs.202413719

**Published:** 2025-02-20

**Authors:** Jeongmin Jang, Junsuk Rho, Hee Jun Shin

**Affiliations:** ^1^ Pohang Accelerator Laboratory POSTECH Pohang 37673 Republic of Korea; ^2^ Department of Mechanical Engineering Pohang University of Science and Technology (POSTECH) Pohang 37673 Republic of Korea; ^3^ Department of Chemical Engineering Pohang University of Science and Technology (POSTECH) Pohang 37673 Republic of Korea; ^4^ Department of Electrical Engineering Pohang University of Science and Technology (POSTECH) Pohang 37673 Republic of Korea; ^5^ POSCO‐POSTECH‐RIST Convergence Research Center for Flat Optics and Metaphotonics Pohang 37673 Republic of Korea; ^6^ National Institute of Nanomaterials Technology (NINT) Pohang 37673 Republic of Korea

**Keywords:** amplitude modulation, pump–probe, THz metasurface, ultrafast all‐optical switching, waveform control

## Abstract

The development of high‐speed and high‐performance optical switches has been a long‐standing issue in the field of photonics. This paper introduces a pioneering time‐resolved spectroscopy‐based approach for realizing photon‐induced ultrafast terahertz (THz) modulation within an electrical split‐ring resonator (SRR) via photoexcitation, rather than relaxation dynamics, in a silicon‐based indirect‐bandgap material. Two competitive effects (shorting of LC circuit and metallization of substrate) occur during photon‐induced THz modulation. The tradeoff between these two effects enables high‐speed optical switching via different time scales of the photoexcitation processes—THz‐optical cooperative effect and phonon‐assisted electron transition. THz‐optical cooperative photoexcitation, causing a shorting effect within the LC circuit, has been observed in the SRR gap, whose size typically exceeds that facilitating impact ionization (IMI). Notably, a remarkably short THz switching time of 1.3 ps has been achieved via only photoexcitation and with a high‐performance transmission intensity modulation depth of over 500%. In addition, active temporal waveform control down to a sub‐cycle pulse has been successfully demonstrated. The proposed approach suggests a new route for constructing high‐speed and efficient THz dynamic photonic devices with potential applications in temporal waveform control.

## Graphical abstract

This study introduces a novel time‐resolved spectroscopy approach enabling ultrafast terahertz (THz) modulation in silicon‐based optical switches. Utilizing photon‐induced processes, it achieves a record THz switching time of 1.3 ps and a modulation depth over 500%. The method demonstrates active sub‐cycle pulse control, offering a new pathway for high‐speed, efficient THz photonic devices.

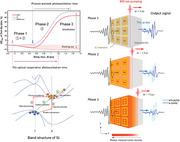

## 1 Introduction

Optical switching plays a crucial role in the realm of active photonic devices, encompassing light generation, manipulation, and detection. In addition, high‐speed optical switches with large modulation depths are in demand for numerous modern scientific and technological applications, such as pulse wavefront shaping,^[^ ^1^, ^2^ ^]^ holography or display,^[^ ^3^, ^4^ ^]^ optical computing,^[^ ^5^, ^6^ ^]^ quantum information processing,^[^ ^7^ ^]^ integrated photonics,^[^ ^8^, ^9^ ^]^ and data encoding.^[^ ^10^ ^]^ Similarly, light modulation in the terahertz (THz) range has garnered considerable attention owing to its potential for waveform control,^[^ ^11^, ^12^ ^]^ high‐speed sensing,^[^ ^13^ ^]^ imaging,^[^ ^14^, ^15^ ^]^ THz filtering,^[^ ^16^ ^]^ and wireless communications systems.^[^ ^17^, ^18^ ^]^ However, despite the recent advances in THz light manipulation, achieving both ultrafast switching speed and large modulation depth, which are the two main figures of merit (FOM), remains challenging.

Metasurfaces, artificially engineered periodic structures of subwavelength meta‐atoms, have revolutionized the field of active photonic devices. Metasurfaces possess unprecedented and versatile light manipulation ability, particularly, in the THz frequency range, where natural materials exhibit limited optical responses. Given these advantageous features, metasurfaces facilitate the fabrication of novel photonic devices. Split‐ring resonators (SRR) are the most extensively studied conventional metastructures, offering high sensitivity to the surrounding substrate environment and serving as perfect platforms for light manipulation, which involves amplitude modulation,^[^ ^19^, ^20^, ^21^, ^22^, ^23^ ^]^ frequency tuning,^[^ ^24^, ^25^, ^26^, ^27^ ^]^ and polarization control using chiral metasurfaces.^[^ ^28^, ^29^, ^30^ ^]^

Many recent studies have focused on enhancing the functionality of SRR metasurfaces by hybridizing them with various dynamic substrate materials, such as semiconductors,^[^ ^31^, ^32^, ^33^ ^]^ graphene,^[^ ^34^, ^35^, ^36^ ^]^ transition metal dichalcogenides (TMDCs),^[^ ^37^, ^38^ ^]^ and phase‐change media.^[^ ^39^, ^40^ ^]^ Specifically, the THz transmission characteristics of these metasurfaces can be modulated by diverse external stimuli, such as voltage bias,^[^ ^41^, ^42^, ^43^ ^]^ thermal effects,^[^ ^44^, ^45^ ^]^ and optical light.^[^ ^46^, ^47^, ^48^, ^49^, ^50^, ^51^, ^52^, ^53^ ^]^ Photon‐induced THz manipulation—all‐optical THz switching—outperforms other methods in achieving ultrafast optical switching because the modulation speed, which exceeds the tuning speeds of electrical or thermal methods (millisecond scale), is governed by the short relaxation time of the photoexcited carriers (picosecond scale). While photoswitching with substantial amplitude modulation has been demonstrated using conventional semiconductor substrates such as Si^[^ ^47^, ^48^ ^]^ and GaAs,^[^ ^49^, ^50^ ^]^ the inherent long relaxation time of these substrates reduces the switching‐off speed and inhibits ultrafast optical switching. Carrier relaxation times can be ideally shortened to ≈10 ps typically by introducing structural defects that promote carrier recombination in dynamic materials (e.g., ErAs nanoislands^[^ ^54^ ^]^ and low‐temperature grown GaAs^[^ ^55^ ^]^). Recently, nonconventional dynamic materials with short carrier relaxation times have been employed to achieve ultrafast switching with extraordinary speeds. For example, ErAs islands embedded inside a GaAs substrate promote carrier recombination and shorten the optical switching time to ≈20 ps.^[^ ^51^ ^]^ Alternately arranged atomic layers in a 2D perovskite substrate induce photoexcited carrier relaxation at ultrafast timescales, achieving a switching time and modulation depth of 20 ps and 93%, respectively.^[^ ^52^ ^]^ Similarly, structural defects in an amorphous germanium substrate promote ultrafast carrier recombination, leading to a remarkable switching time of 17 ps with a modulation depth of 90%.^[^ ^53^ ^]^ In addition, TMDCs, characterized by optical switching times of the order of 10–100 ps, outperform conventional semiconductors in reducing carrier recombination time.^[^ ^37^, ^38^ ^]^ However, despite recent advancements, the current defect introduction approach remains limited to switching times within the range of several picoseconds.

Pulse waveform control is a critical aspect of optical manipulation, with significant efforts devoted to shaping pulses within few picoseconds or even sub‐picosecond timescales. Recent studies have demonstrated waveform control over a wide spectral range (visible, infrared, and THz regions) via Fourier transform waveform control,^[^ ^56^, ^57^ ^]^ pulse shaping diffractive layers,^[^ ^58^ ^]^ and cascaded intrapulse difference‐frequency generation.^[^ ^59^ ^]^ These techniques involve tuning of the relative phase of the spectral components and synthesizing them into a shape‐modified pulse. This coherent pulse synthesis is considered the best strategy for realizing specific waveform control, such as shortening the pulse duration to beyond the sub‐cycle limit, which is of paramount interest from both fundamental and application perspectives.^[^ ^59^, ^60^ ^]^ Most methods for pulse waveform control developed to date employ passive‐type pulse manipulation techniques, thereby enabling slow manipulation. Notably, ultrafast active‐pulse‐waveform control has emerged as a promising tool to overcome these limitations, and ultrafast temporal optical switching, within timescales shorter than the pulse duration, may offer a highly efficient and straightforward route to overcome the sub‐cycle limit.

The primary structural feature of SRRs for THz switching is the semiconductor‐based capacitive gap, with gap size determining resonance frequency. Photodoping gradually enhances gap conductivity, modulating the THz signal over time. Recent research indicates that in nanogap structures, the THz field can be enhanced by over 100 times, enabling THz self‐modulation.^[^ ^61^, ^62^ ^]^ This enhanced THz field accelerates conduction‐band electrons, generating electron–hole (e–h) pairs via impact ionization (IMI), differing from interband transition driven by optical pumping.^[^ ^63^, ^64^ ^]^ While amplifying the THz field through a narrow SRR gap offers various advantages, it also introduces critical disadvantages. The intense electric field increases conduction and dielectric losses and heightens the risk of electrical breakdown, compromising structural reliability.^[^ ^44^ ^]^ For optical switching, excessive THz field enhancement in narrow SRR gaps can cause premature circuit shorting and weaken LC resonance (L: inductor; C: capacitor),^[^ ^43^ ^]^ reducing the optical pump‐induced amplitude modulation depth. Conversely, a wider SRR gap could mitigate these disadvantages; however, as the capacitive gap of the SRR widens to suppress IMI, a novel physical phenomenon is required to induce the shorting effect across the gap for THz modulation.

In this study, we developed an approach for ultrafast THz manipulation using an SRR metasurface, with a several micron‐wide capacitive gap on an indirect‐bandgap Si substrate, thus achieving rapid and large‐modulation‐depth THz switching. Photoexcited free carriers in the Si substrate enhance THz transmission by shorting the split‐ring resonance, while optical pumping reduces THz transmission via bulk Si wafer metallization. These effects (capacitive gap shorting^[^ ^48^, ^49^ ^]^ and metallization^[^ ^65^, ^66^ ^]^) have been observed independently; however, their ultrashort timescale sequence offers a novel approach to ultrafast optical switching. Utilizing the “photoexcitation process” instead of “relaxation dynamics” leverages the faster electron excitation timescales, enabling rapid switching speeds. Our experiments show that overlapping THz pulses and optical pumping create a “THz‐optical cooperative photoexcitation” mechanism. A single optical pump induces a sequential two‐step photodoping process across the metasurface, with activity in the SRR capacitive gap occurring hundreds of femtoseconds before the phonon‐assisted interband transition in the rest of the indirect‐bandgap substrate.

## 2 Results

### 2.1 All‐Optical THz Switching via Ultrafast Time‐Variant Photodoping of an SRR

**Figure 1:**
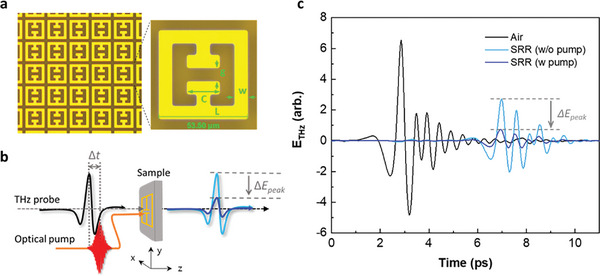
Schematic of OPTP experiment on SRR metamaterial. a) Optical image of a periodic array of the THz SRR structure fabricated on a 500‐µm‐thick bulk Si substrate. Geometric parameters are described in the Methods section. b) Schematic of our OPTP experiment. The THz probe pulse is delayed (*Δt*) relative to the optical pump to explore transmission modulation in the photodoping process. (Red pulse: illuminating 800‐nm pump; black pulse: incident THz pulse; light and deep blue pulses: THz transmission without and with the pumping, respectively. c, 800‐nm pumping gradually reduces the THz transmission of the metamaterial (blue data: measured at a quasi‐steady state after pumping, phase 3 in **Figure** **2**).

A schematic of the optical pump–THz probe (OPTP) experiment performed on an electric SRR structure is shown in **Figure** **1**. This component is formed by combining two individual SRRs on the split‐gap side. The integration of the two rings contributes to an inductance and the split‐gap contributes to a capacitance, resulting in the generation of LC resonance (Lorentzian shaped). As the photon energy of the 800‐nm pump (1.55 eV) exceeds the bandgap of the Si substrate in the metamaterial (*E_g_ =* 1.12 eV^[^ ^67^ ^]^), electrons are excited from the valence band to the conduction band. The transition from semiconductor to metal improves the conductivity of the substrate (Figure S1, Supporting Information), thereby leading to a decrease in THz transmission (*ΔE_peak_* in Figure 1c).

**Figure 2:**
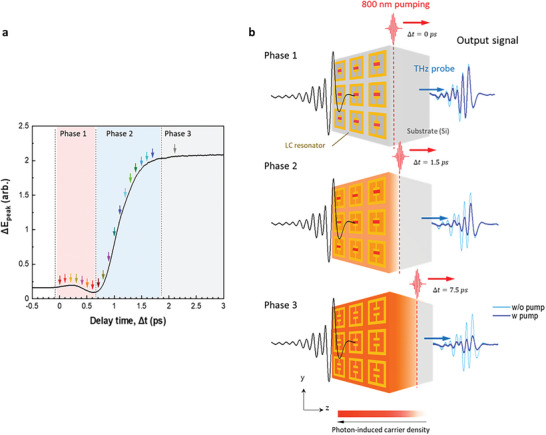
Schematic of OPTP experiment with various probe delay times (*Δt*). a) Change in the THz main peak as a function of different delay times (note that $\Delta E_{peak}\left(\Delta t\right)=|E_{peak}^{no\;pump}\left|\;-\right|E_{peak}^{pump}\left(\Delta t\right)|$). The red, blue, and gray regions represent three different trends, namely, phases 1, 2, and 3, respectively. b) Illustration showing that different probe delay times (left) produce dissimilar THz modulations (right). *Δt* = 0 reflects the temporal overlap of the pump and probe peaks. As the delay time (*Δt*) increases (phase 1 → 3), the incident probe experiences a higher photon‐induced carrier density at the Si substrate. The optical pump beam uniformly illuminates the metasurface; however, carrier photodoping occurs first at the SRR capacitive gap due to the “THz‐optical cooperative photoexcitation” effect (phase 1), followed by metallization across the remaining parts of the substrate (phases 2 and 3). Detailed explanation is provided in the Discussion section. These three sequential steps of local doping manifest as real‐time changes in the THz pulse profile (**Figure** **4**a).

Figure 2a shows the optical pump‐induced change of THz main peak (*ΔE_peak_* is defined in Figure 1c) at various delay times. The observation that all electronic transitions occur within a significantly shorter duration on the photoexcitation timescale (≈2 ps) than in the relaxation regime (>1 ns^[^ ^65^, ^66^ ^]^) has prompted a new approach. That is, utilizing this timing is more favorable for ultrafast THz modulation. Three different types of transmission changes were observed: the initiation of change (phase 1), rise time (phase 2), and quasi‐steady state (phase 3). Because the timescales of the excitation process and the pulse duration of the THz probe are comparable, the probe pulse penetrates the sample where the carrier density changes in real time. Consequently, the delay significantly influences the shape of the transmitted THz pulse (Figure 2b). For instance, in the case of a long delay between the pump and probe, the THz probe encounters an already‐excited electron state, and most parts of the pulse are modified (phase 3 in Figure 2b). However, during a short delay, a subtle change occurred in the pulse head (main peak), and this was followed by a gradual increase in this change (pulse tail) (Phase 1, Figure 2b).

### 2.2 Ultrafast Speed and Large Modulation Depth of All‐Optical Switching

**Figure 3:**
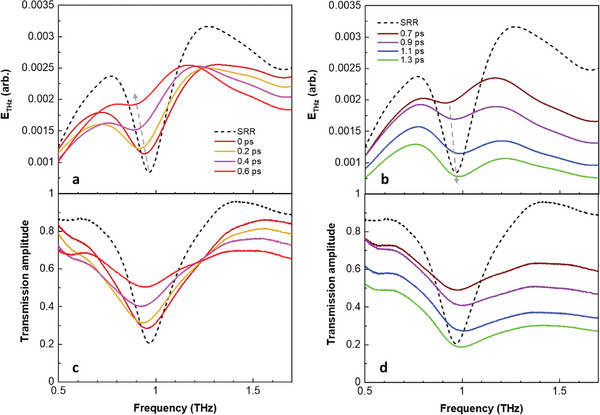
Photon‐induced ultrafast THz amplitude modulation. Transmitted THz field spectra in a) phase 1 and b) phase 2. Transmission amplitude in pumping c) phase 1 and d) phase 2. Transmission amplitude is obtained from $t\;\left(\omega \right)=\frac{E_{SRR}\left(\omega \right)}{E_{Si}\left(\omega \right)}$, where *E_SRR_* (ω) and *E_Si_* (ω) are the measured THz spectra of the sample and Si substrate, respectively. The colors of the spectral lines follow the arrow in Figure 2a. This system demonstrates the operation of photon‐induced THz switching within 1.3 ps. In phase 1, the gradual disappearance of the split‐ring resonance is observed due to pump‐induced photocarriers in the Si substrate. In phase 2, increased photodoping gives rise to the excessive metallization of the Si substrate, which consequently results in a reduction in THz transmission. These highlight competing effects between Lorentzian LC resonance disappearance and substrate metallization, thereby enabling ultrafast THz switching.

Interestingly, the two distinct delay phases yield opposing THz amplitude modulations, suggesting the realization of a new concept optical switching. In the short interval between the pump and probe (phase 1), there was an enhancement in the THz transmission at the Lorentzian LC resonance (≈0.97 THz) (**Figure** **3**a,c). Conversely, for the longer pump–probe delay (phase 2), the transmission was reduced (Figure 3b,d). The complete data are presented in Figure S2 (Supporting Information). In the experiment with the Si substrate, a consistent reduction in transmission was observed for all delay times (Figure S3, Supporting Information). This result indicates that optical pump‐induced free carriers degrade the electrical LC resonance and ultimately activate the “THz switching on.” The first half of the photoexcitation (0 < *t* < 0.6 ps) enhances the transmission of the THz probe pulse, whereas the second half (0.6 < *t* < 1.3 ps) diminishes the transmission of the THz probe pulse. These findings confirm the realization of photon‐induced ultrafast switching with a remarkably short switching time of 1.3 ps, which is a significant improvement over that achieved in previous studies,^[^ ^37^, ^38^, ^50^, ^51^, ^52^, ^53^ ^]^ along with the achievement of rapid and efficient modulation. During switching on, a maximum amplitude modulation depth ($\frac{t_{on}-t_{off}}{t_{off}}\times 100\%$) of 144% and intensity modulation depth ($\frac{t_{on}^{2}-t_{off}^{2}}{t_{off}^{2}}\times 100\%$) of 500% were achieved, as calculated in previous studies.^[^ ^68^, ^79^ ^]^ This modulation depth also suggests a remarkable enhancement over that achieved using previous photon‐induced SRR modulators.^[^ ^50^, ^52^, ^53^, ^68^, ^69^ ^]^

The disappearance of the split‐ring resonance characteristics in phase 1 (Figure 3a) reproduced the previous results of photoexcitation in Si‐based^[^ ^48^ ^]^ or GaAs‐based SRRs,^[^ ^49^ ^]^ doping effect in GaAs‐based SRRs,^[^ ^41^ ^]^ and thermal excitation in VO_2_‐based SRRs.^[^ ^44^ ^]^ This suggests that the 800‐nm pumping step metallizes the Si substrate in the gap by inducing photodoping, thereby gradually shorting the capacitive gap. Subsequently, a sufficiently metallized bulk Si substrate reduces the THz transmittance across all frequencies (Figure 3b), consistent with the degradation of THz transmission upon exposure of the bulk Si substrate to a sufficient 800‐nm pump energy.^[^ ^65^, ^66^ ^]^ Our findings indicate that the two coexisting yet competing effects (vanishing Lorentzian LC resonance and substrate metallization) play a crucial role in the THz modulation of semiconductor‐based SRR metasurfaces and that ultrafast THz switching can be realized by leveraging this tradeoff relationship.

### 2.3 Time‐Resolved Ultrafast Switching

**Figure 4:**
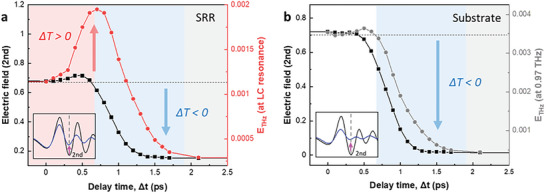
Analysis of the time‐domain pulses and the tradeoff relationship in THz transmission. a) A comparison of pump‐induced change in the magnitude of the second dip in the time‐domain pulse (depicted by black scatter line, positions of second dip is shown in the inset) with the corresponding spectral data (red scatter line) in the case of SRR is illustrated. This graph shows a tradeoff relationship wherein two competing effects exist for THz transmission near the Lorentzian LC resonant frequency of 0.97 THz. The red, blue, and gray regions correspond to phases 1, 2, and 3, respectively. During the initial moments of optical pumping (phase 1), photodoping in the capacitive gap shorts out the LC resonance of the SRR circuit, thereby enhancing transmittance around the resonance (right red axis), which correlates with the strengthening of the second dip in the transmitted pulse (left black axis). In the latter moments of photoexcitation (phase 2), the abundantly generated carriers in the residual sample region (≈9 times larger area than the capacitive gap; see Figure 1a) excessively metalize the Si substrate. This effect dominates over the shorting effect and reduces the transmittance. b) By contrast, in the Si substrate, a consistent reduction in transmission is observed during the delay time.

To understand the origin of the tradeoff (shorting of LC circuit vs metallization of substrate), a detailed analysis of the temporal evolution of the time‐domain pulse was conducted (see Figure 4). Notably, the variation in the THz main peak observed in the OPTP experiment (Figure 2a) is primarily attributed to frequency components near 0.75 THz, rather than to those linked to the Lorentzian LC resonance frequency (Figure S4, Supporting Information). The analysis revealed that the second dip of the THz pulse is considered to primarily contain Lorentzian LC resonance component near 0.97 THz. The magnitude of the pulse second dip is enhanced rather than reduced by optical pumping (Figure S5a inset, Supporting Information), aligning with the transmission changes in the LC resonance (Figure 4a: black vs red line). The strong correlation between these two parameters suggests that the second pulse dip contains predominantly Lorentzian LC resonance components. These findings suggest the following three key points:

1. Optical pumping improves the transmission only in the SRR structure and not in the Si substrate (Figure 4a versus 4b), indicating that the origin of “switching on” is a photon‐induced electrical change in the LC circuit.
2. Transmission improves only when the probe pulse traverses the SRR structure immediately after pumping (phase 1: within 0.6 ps). This result suggests that in the initial moments of pumping, the shorting effect of the capacitive gap dominates over the metallization effect of the remaining substrate (details in Figure 7a and Section 3).
3. Two competitive effects modulate the THz transmission. Pump‐induced photoconductivity reduces the transmission by metallizing the semiconductor substrate while improving it by shorting the Lorentzian LC resonance. This tradeoff relationship suggests that active THz switching can be realized in the photoexcitation regime.

**Figure 5:**
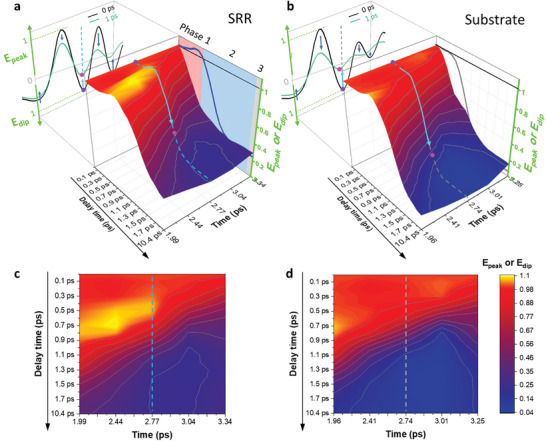
Pump‐induced modulation of the THz pulse and the underlying mechanism of ultrafast THz switching in bulk Si‐based SRR. The pump‐induced change of the THz pulse amplitude and corresponding 3D plot for the SRR (a) and Si substrate (b), respectively, are illustrated. The pulse profiles indicate a reduction in transmission with probe delay time (black line: *Δt* = 0 ps, cyan: *Δt* = 1 ps). The 3D contour plot shows temporal change in the magnitudes of the two main pulse peaks and the three main dips relative to the delay time, with values normalized to the data at the zero‐delay time (*Δt* = 0 ps). A vertical dashed line marks the location of the second dip, at 2.77 ps for the SRR (a), where the Lorentzian LC resonance information is mainly included and at 2.74 ps for the Si substrate (b). Two points (purple circle: *Δt* = 0 ps, pink pentagon: *Δt* = 1 ps) and a light blue arrow are presented to enhance visual understanding. The value of the time‐dependent second dip is projected onto the right plane. Regions denoted in red, blue, and gray represent phase 1, 2, and 3 respectively. a) During the initial moments of pumping (phase 1), the magnitude of the pulse tail (*t* > 2.9 ps) is drastically reduced (steep negative slope in the 3D plot). However, at the pulse head (second dip, *t* = 2.77 ps), enhanced transmission is observed remarkably (bright yellow region and projection line on the right red plane). Such enhancement gradually disappears in the successive suppression effect caused by the excessive metallization of the Si substrate (negative slope in the 3D plot and projection line on the right blue plane). These highlight a turning over (at approximately *Δt* = 0.6 ps) in the dominant effect between two competing effects modulating transmission near the Lorentzian LC resonant frequency of the SRR. By contrast, in the case of the Si substrate (b), a monotonic reduction in transmission was observed for all pulse peaks and dips. c,d) show corresponding 2D plots for the SRR and Si substrate, respectively.

**Figure** **5** shows the turnover of the dominant effect among the two competing influences in the Lorentzian LC resonance as the delay time changes. When the probe pulse passed immediately after pumping, the tail was primarily weakened (t > 2.9 ps in Figure 5a). As the delay between the pump and the probe increased, the degradation in the transmission of the Si substrate gradually extended to the entire frequency range (Figure 5b). However, in the measurement of the SRR, a notable improvement in the transmission in the second dip was observed in phase 1 (bright yellow region and projection line in the right red plane in Figure 5a), where the LC resonance information is contained (Figure 4a). As the pump–probe delay increases, this enhancement gradually diminishes as the significant degradation caused by the metallization of the semiconductor substrate becomes more pronounced (projection line in the blue plane in Figure 5a).

### 2.4 Active THz Temporal Waveform Control

**Figure 6:**
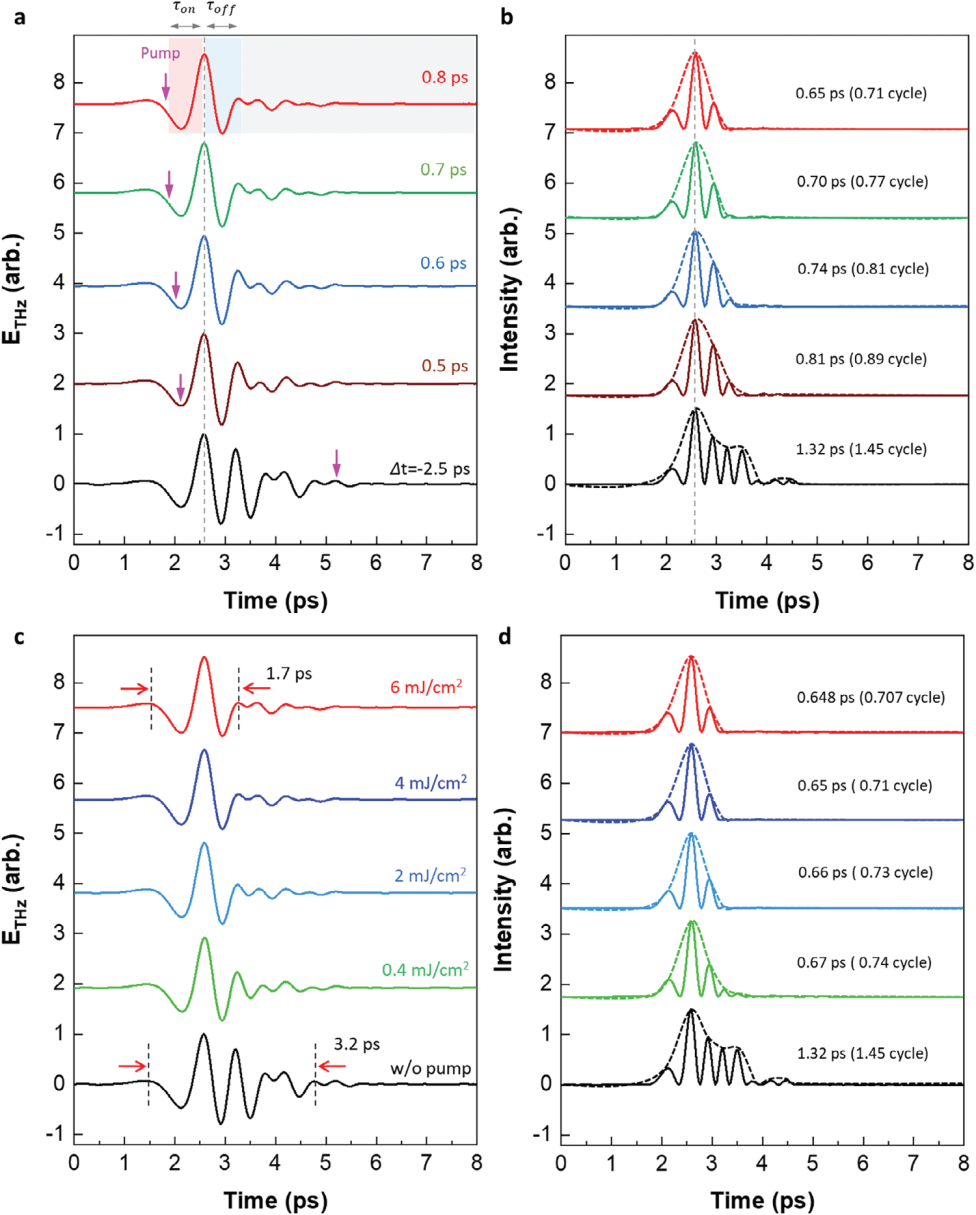
Active THz waveform control: Switching from few‐cycle to sub‐cycle THz pulses. a) Temporal control of THz pulse shape with various pump–probe delay time (*Δt*) at optical pump fluence 4 mJ cm^−2^. Pink vertical arrow and gray dashed line represents timing of optical pump and position of main peak, respectively. Due to causality, the THz probe pulse shape is modified only when preceded by the pump pulse (*Δt* > 0). *τ_on_* (0.6 ps) and *τ_off_* (0.7 ps) denote period of switching on and off, respectively. Because our switching‐off time (0.7 ps) is much shorter than the width of the THz pulse profile (≈ 3.2 ps), effective conversion from a few‐cycle pulse to sub‐cycle pulse is realized by enhancing the main part (switching on, red area) and suppressing the following multitails (switching off, blue, and gray area). b) Corresponding intensity envelopes, FWHM pulse duration, number of pulse cycles are calculated at the spectral centroid (≈1.1 THz). c) Optical pump fluence‐dependent THz waveform control at a delay time of 0.8 ps. The higher the pump energy, the better the waveform control, and above 4 mJ cm^−2^, the pulse shape is saturated to a sub‐cycle within 1.7 ps, which is very close to our switch operating time (≈ 1.3 ps). d) Corresponding intensity envelopes and number of pulse cycles are calculated at the spectral centroid (≈1.1 THz). The main peaks of all the pulses are normalized.

Since the invention of the laser, consistent efforts have been made to shorten the light pulse duration and reach, even break through, the sub‐cycle limit.^[^ ^60^ ^]^ Ultrashort THz pulses are technically advantageous for various applications, including investigation of novel ultrafast and nonlinear phenomena such as molecular alignment, ultrafast energy transfer, and excitation of spin state or crystal lattice.^[^ ^59^, ^70^, ^71^, ^72^, ^73^, ^74^ ^]^ In this study, we successfully overcame the sub‐cycle THz pulse limit through temporal optical switching. **Figure** **6** illustrates modulation from few‐cycle to sub‐cycle THz pulses using our ultrafast all‐optical switching method. Figure 6a presents the temporal change in the pulse waveform with various pump–probe delay times. Due to causality, the THz probe pulse shape is modified only when preceded by the pump pulse (*Δt* > 0 ps). Because our switching‐off time (*τ_off_* = 0.7 ps) is considerably shorter than the THz pulse profile (3.2 ps), efficient conversion from few‐cycle to sub‐cycle pulses is realized by enhancing the amplitude of the main peak of the pulse (switching on, red area in Figure 6a) and suppressing the following multitails (switching off, blue and gray area in Figure 6a). The full width at half maximum (FWHM) duration and number of pulse cycles are defined based on the intensity envelope^[^ ^59^ ^]^ (Figure 6b). By matching the optical switching with the pulse's main peak (at *Δt* = 0.8 ps), effective manipulation to a sub‐cycle pulse (0.7 cycle) has been achieved. In addition, pump‐fluence‐dependent waveform control has been investigated. When the pump energy is increased, the pulse waveform duration can be shortened. However, if the pump fluence exceeds 4 mJ cm^−2^, then the pulse shape is saturated to a 0.71‐cycle pulse (Figure 6c,d). Our approach demonstrating active temporal waveform control is a significant advancement over the previous passive‐type waveform control methods.^[^ ^56^, ^57^, ^58^, ^59^ ^]^

## 3 Discussion

To verify the reliability of our experimental setup, we examined the changes in the photoconductivity and carrier density of the Si wafer as functions of the pump fluence. The conductivity of the Si wafer increased with the pump fluence, and the experimental data reproduced previous result^[^ ^75^ ^]^ (Figure S6, Supporting Information). The photocarrier density is analyzed from the conductivity shows an inverse relationship with the changes in the THz pulse peak, which is reasonable. Additionally, the saturation behavior of the carrier density at a high pump fluence reproduced the results of previous study.^[^ ^66^ ^]^ Considering that the resistivity of our Si wafer (10 kΩ) is ten times higher than the resistivity (1 kΩ) used in previous studies, the observation of nearly 10 times lower carrier density is reasonable (Figure S7, Supporting Information).

To elucidate the time‐resolved mechanism of ultrafast THz modulation in Si‐based SRR metamaterials, several physical scenarios have been proposed herein. When THz waves pass through an SRR metamaterial, resonant frequencies are generated owing to THz plasmons. During this process, the THz electric field is strongly enhanced in the gap of the SRR. The amplified THz electric field is not uniformly distributed across the gap; instead, its intensity decreases toward the center of the gap. At the edges of the gap, the THz electric field is amplified by more than 90 times compared with the incident field (see Figure S8, Supporting Information). The incident THz electric field, generated through optical rectification with a ZnTe nonlinear crystal, is approximately few kV/cm. Therefore, the amplified THz electric field at the gap edges exceeds 500 kV cm^−1^.

**Figure 7:**
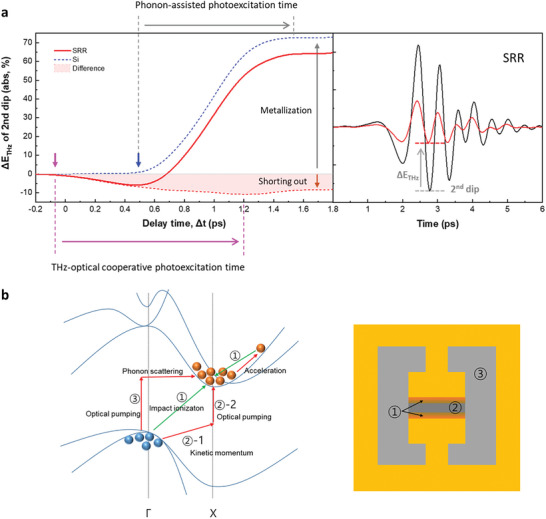
Time‐resolved dynamics of THz pulses and carrier transition processes in SRR metamaterials due to IMI, THz‐optical cooperative photoexcitation, and phonon‐assisted transition. a) Temporal change in the magnitude of the second THz pulse dip for the SRR (red solid line) and Si substrate (blue dashed line) as a function of probe delay time (note that $\Delta E_{THz}\;\left(\Delta t\right)=\frac{\left(|E_{dip}^{no\;pump}\left|-\right|E_{dip}^{pump}\left(\Delta t\right)|\right)}{|E_{dip}^{no\;pump}|}\;\times 100\%$); the difference is also shown (red dashed line). THz field induces IMI, generating e–h pairs, and THz‐optical cooperative photoexcitation occurs via 800‐nm optical pumping, creating a shorting effect in the SRR gap (0–0.5 ps). Phonons transfer energy to carriers, causing X‐valley transitions, while IMI THz‐optical cooperative photoexcitation persists (0.5–1.2 ps). The IMI and photon‐assisted transition processes are completed, and carrier doping saturates (>1.2 ps). The difference and Si data represent the temporal shorting and metallization behaviors, respectively. b) Carrier transitions in Si band structure (left) and corresponding regions in the SRR structure (right). A strong electric field accelerates conduction‐band electrons, inducing IMI and generating e–h pairs at the gap edges (process ①). The carrier concentration at the gap edges reaches ≈1.6 × 10¹⁶ cm⁻^3^. The photon‐induced electron transition to the X‐valley via an 800 nm optical beam also occurred same time (process ②). This transition occurs rapidly due to minimal phonon influence, and a shorting effect is observed in the metamaterial gap. In areas outside the SRR gap, the optical beam forms hot carriers that generate optical phonons. As optical pumping continues, optical phonons transfer energy to valence‐band carriers, inducing further transitions to the X‐valley (process ③).

To analyze carrier dynamics in the silicon substrate of the SRR in more detail, we consider two types of regions: one with a short circuit at the gap of the SRR and the other with metallization on the silicon surface (**Figure** **7**).

1. 0–0.5 ps: Considering the phenomenon of IMI, in which a strong electric field accelerates conduction‐band electrons to collide with valence‐band electrons, generating e–h pairs, we calculated the carrier concentration induced by IMI as a function of electric field strength (see Figures S8 and S9, Supporting Information). This process is governed by the principles of energy and momentum conservation and can occur only if the electron kinetic energy exceeds the IMI threshold energy of 3.6 eV in silicon,^[^ ^76^ ^]^ rather than the bandgap energy of silicon, which is 1.12 eV. We calculated the kinetic energy of the electron under THz field strength (see Figure S9, Supporting Information). Near the edge of the gap, the electron's kinetic energy is sufficient to enhance IMI process. Once the electron achieves this energy level, it can generate an e–h pair (process ①), as shown in Figure b (left). Following this, the original electron, which loses energy during the creation of the e–h pair, and the newly formed e–h pair can continue to gain energy from the applied electric field. This sequential process can be sustained until the THz‐pulse‐induced electric field remains sufficiently strong. Even with relatively few IMI events, this mechanism has the potential to produce a significant number of e–h pairs, amplifying the overall effect. The carrier concentration at the gap edges was estimated to be ≈1.6 × 10¹⁶ cm⁻^3^ (see Figure S9, Supporting Information). However, because the electric field decreases in strength as it moves toward the center of the gap, and the kinetic energy of the electrons is insufficient to exceed the IMI threshold energy of silicon, IMI is suppressed. Therefore, the carrier concentration remains at its initial value (see Figure S9, Supporting Information). Although the kinetic energy of the electrons is lower than the IMI threshold energy, conduction‐band electrons, accelerated by a moderately amplified electric field, induce momentum changes in valence‐band electrons. This enables the electron to transit to the X‐valley conduction band via the 800‐nm optical beam (process ② termed THz‐optical cooperative photoexcitation), as shown in Figure b (left). At this stage, due to the minimal influence of phonons, the electron transition induced by the optical beam proceeds more rapidly. The behavior of the second dip of the THz pulse in the SRR, as shown in Figure 7a, reveals that the generated carriers contribute to change in conductivity. Thus, a shorting effect can occur in the gap of the SRR metamaterial prior to the intervalley transition caused by interactions with phonons generated by the 800‐nm optical beam pumping. In areas of the silicon substrate other than the SRR gap, the 800‐nm photon transfers energy to conduction‐band electrons, forming hot carriers (< 0.1 ps). These hot carriers then generate optical phonons (< 0.5 ps). Because silicon is an indirect‐bandgap material, the timescale of interband electron transitions is slower than that of direct‐bandgap materials due to the requirement for phonon energy transfer to electrons. Consequently, IMI and THz‐optical cooperative photoexcitation can occur before phonon‐assisted band‐to‐band transitions. In addition, shorting at the gap can occur through two processes: IMI and THz‐optical cooperative photoexcitation. These phenomena occur exclusively at the interface between the SRR structure and the substrate when the optical pump beam is directed onto the SRR patterns (see backside optical pumping in Figure S10, Supporting Information; no shorting effect is observed in this case).
2. 0.5–1.2 ps: At the onset of 800‐nm optical pumping, optical phonons transfer energy to the carriers in the valence band, inducing a transition of hot carriers to the X‐valley (>500 fs) (process ③). At this stage, the phonon‐induced carrier doping effect becomes dominant and is attributed to the photoinduced carrier doping in the silicon substrate. Consequently, the temporal carrier doping can be ascribed to the effect of pump‐fluence‐dependent doping on the steady‐state THz transmission of the SRR metasurface (Figure S11, Supporting Information), while the IMI THz‐optical cooperative carrier transitions persist.
3. >1.2 ps: At this stage, IMI and photon‐assisted carrier transitions are completed, and the photoinduced carrier doping saturates.

We believe that process 1) (0–0.5 ps) plays a key role in silicon‐based SRR metamaterials. The THz electric field accelerates the electrons in the conduction band, thus changing their kinetic momentum. Subsequently, the valence‐band electrons undergo e–e scattering with the conduction‐band electrons, thereby occupying higher‐energy states and transitioning to a nonequilibrium distribution in the *k*‐space (②‐1 in Figure 7). Thus, the probability of transitions to the conduction band, induced to an 800‐nm optical beam without the assistance of phonons, increases (②‐2). Furthermore, the observation of process 1) within 0.5 ps can be attributed to the indirect‐bandgap characteristics of silicon. In the case of a direct bandgap, an optical source induces transitions within ultrashort timescales, making it difficult to observe process 1) distinctly. However, in silicon, which has an indirect bandgap, transitions due to optical pumping involve a series of processes, that is, hot carrier generation, hot carrier–lattice interactions, phonon generation, and changes in electron momentum due to phonons, which lengthen the transition time compared to that observed in the case of direct‐bandgap materials. In our silicon‐based SRR metamaterial, the timescale of the shorting effect caused by the THz electric field was shorter than that in which the valence‐band electrons transitioned to the conduction band via phonon interactions after optical pumping. Thus, the shorting effect within the metamaterial gap could be clearly visualized.

THz amplitude modulation using ultrafast all‐optical switching has been extensively studied (**Table** **1**). Recent studies have primarily focused on integrating ultrathin, ultrafast photoactive materials with metasurfaces, which hold significant potential for applications in flexible and miniaturized THz photonic devices,^[^ ^77^, ^78^, ^79^ ^]^ ultrafast wireless THz communication,^[^ ^38^, ^52^, ^80^ ^]^ and switchable sensor, filter, modulator.^[^ ^37^ ^]^ In this study, we aimed to achieve a large modulation depth simultaneously with a switching time shorter than the pulse duration (≈3 ps) for effective pulse shape modulation. Fano and quasi‐bound state in the continuum (QBIC) resonances are highly sensitive to temporal changes in photoactive materials, and are thus ideal for ultrafast THz modulation. However, their sharp and asymmetric spectral features hinder their application in active pulse shaping, especially for pulses with moderate or broadband widths (e.g., THz time‐domain spectroscopy: 0.1–2.0 THz). Lorentzian LC resonance, with its symmetric profile, allows for easier transition between band‐pass and band‐stop states through active switching, enabling temporal pulse modulation over a wide frequency range. The findings of this study highlight the two key FOM—modulation depth and switching time—as crucial parameters for effective active sub‐cycle pulse modulation.

**Table 1:** All‐optical switching‐based THz amplitude modulators and FOM.

Design	Photoactive materials	Method	Mode	Modulation depth [%]	Switching time [ps]	Advantage	References
ASRR	2D perovskite (60 nm)	Optical pumping	Fano	90	20	Flexible, stable	[52]
ASRR	SnSe_2_ (10 nm)	Optical pumping	Fano	91	15	Low power, stable	[38]
ASRR	Ge (310 nm)	Optical pumping	Fano	90	17	Flexible	[53]
EIT	Bi_2_Se_3_ (20 nm)	Optical pumping	EIT	31	9.5	Ultrathin	[81]
2D materials	Graphene on SU‐8	Optical pumping	Coherent interference	40	2.8	Ultrathin	[78]
ASRR	Ge (500 nm)	Optical pumping	QBIC	75, 200*	7	Flexible, low‐loss	[79]
ASRR	Ge (50 nm)	Optical pumping	Fano	85	2.5	Flexible, low‐loss, low power	[77]
ASRR	MoS_2_ (400 nm)	Optical pumping	Fano	100	100	Low power	[37]
**SRR**	**Si (500 µm)**	**THz‐optical cooperative photoexcitation**	**Lorentzian LC**	144, 500*	**1.3**	**Stable, low cost, facile fabrication**	**This work**

Note: ASRR and EIT represents the Asymmetric SRR and Electromagnetically Induced Transparency, respectively. In the column for modulation depth (%), the numbers marked with * represent intensity modulation, and the other values correspond to amplitude modulation.

This study primarily focuses on achieving high performance in the two key FOM within an SRR metasurface and their application to active sub‐cycle pulse modulation, facilitated by the “THz‐optical cooperative photoexcitation” process in the SRR gap. Optical pumping significantly modulates the LC circuit characteristics (Figure 3). This sub‐cycle THz pulse system shows potential for high temporal resolution biosensing, imaging, and spectroscopy. However, practical application requires improved transmission (Figure S12, Supporting Information). Future studies should focus on reducing substrate loss using thin semiconductor films instead of bulk wafers.

## 4 Conclusion

In this study, we demonstrated ultrafast optical switching through a proposed mechanism for photon‐induced shorting at LC circuit and metallization in bulk Si‐based SRRs. Remarkably, an unprecedented switching time of 1.3 ps is achieved using an indirect‐bandgap semiconductor (Si). The shorting in the LC circuit can be explained via the processes of IMI and THz‐optical cooperative photoexcitation, and the metallization of the silicon substrate induced by 800‐nm optical pumping is attributed to phonon‐assisted electron transition. Our ultrafast optical switch can also facilitate temporal active waveform control. Because the switching time is much shorter than the THz pulse width, we realized efficient manipulation to sub‐cycle pulses (≈ 0.7 cycle). This type of ultrafast switching mechanism can potentially transform the photonic research landscape by facilitating the development of high‐speed THz photonic devices and enabling THz waveform control into the sub‐cycle pulse domain; these features are indispensable for manipulating ultrafast and nonlinear phenomena.

## 5 Experimental Section

### Sample Fabrication

The THz metamaterial was fabricated to have a so‐called electric SRR pattern. The metamaterial has a metallic array consisting of two square rings with a single micro‐split gap structure. A polyimide (PI) solution was spin‐coated onto the 500‐µm‐thick high resistive Si wafer (p‐type, 10 kΩ) at 2500 rpm. The PI‐coated Si wafer was baked at 110 °C, for 3 min. Au pattern was fabricated on the PI/Si wafer by photo‐lithography and lift‐off process. The PI/Si wafer substrate was coated with photoresist at 3000 rpm for 40 s and soft baked at 95 °C for 90 s. Subsequently, the substrate was exposed to ultraviolet light for 3 s, followed by hard baking at 110 °C for 3 min and another 30‐s‐long ultraviolet‐light exposure step. After the development of photoresist, a layer of Ti (30 nm)/Au (100 nm) was deposited onto the PI/Si wafer. In this metamaterial metallic array, the designed periodicity, full length (L), capacitor length (C), gap (g), width (w) were 61.5, 53.5, 19.5, 8.5, and 8.0 µm, respectively. The schematic of the metamaterial is shown in Figure 1a.

### OPTP Experiment

For the photon‐induced time‐varying THz modulation, we used an OPTP setup at the fs‐THz beamline of the Pohang Accelerator Laboratory in the Republic of Korea. The optical pump pulse comes from a Ti: sapphire regenerative amplifier (Coherent, Legend Elite) with a pulse duration of 120 fs at a central wavelength of 800 nm and a repetition rate of 1 kHz. An 800‐nm pulse with a fluence of 0–6 mJ cm^−2^ was illuminated on the SRR sample to induce photocarriers in the Si substrate and modulate the THz transmission at the LC resonance frequency (Figures 1, 2, 3, 4, 5: 0.4 mJ cm^−2^, Figure 6: 0–6 mJ cm^−2^). For the THz time‐domain spectroscopy (TDS), an electro‐optic sampling method with ZnTe crystals was used. In the THz TDS, the data acquisition time (or integration time) for the lock‐in amplifier and the settling time at stop position of the motorized delay stage are 100 and 500 milliseconds, respectively. The OPTP experimental scheme is described elsewhere.^[^ ^82^ ^]^

## Conflict of Interest

The authors declare no conflict of interest.

## Supporting information



Supporting Information

## Data Availability

The data that support the findings of this study are available from the corresponding author upon reasonable request.
