# Supplementary material for: Ultrafast All‐Optical Switching and Active Sub‐Cycle Waveform Control via Time‐Variant Photodoping of Terahertz Metasurfaces
Source: Adv Sci (Weinh). 2025 Feb 20;12(14):2413719. doi: 10.1002/advs.202413719 (PMC11984866; doi:10.1002/advs.202413719)
Supplement: Supplementary file 1 — Supporting Information [file ADVS-12-2413719-s001.docx]

**Supporting Information**

**Ultrafast All-optical Switching and Active Sub-cycle Waveform Control via Time-variant Photodoping of Terahertz Metasurfaces**

Jeongmin Jang^1^, Junsuk Rho*^2,3,4,5^, and Hee Jun Shin*^1^

^1^Pohang Accelerator Laboratory, POSTECH, Pohang 37673, Republic of Korea

^2^Department of Mechanical Engineering, Pohang University of Science and Technology (POSTECH), Pohang 37673, Republic of Korea

^3^Department of Chemical Engineering, Pohang University of Science and Technology (POSTECH), Pohang 37673, Republic of Korea

^4^Department of Electrical Engineering, Pohang University of Science and Technology (POSTECH), Pohang 37673, Republic of Korea

^5^POSCO-POSTECH-RIST Convergence Research Center for Flat Optics and Metaphotonics, Pohang 37673, Republic of Korea

*Corresponding author: shinhj@postech.ac.kr (Hee Jun Shin); jsrho@postech.ac.kr (Junsuk Rho)


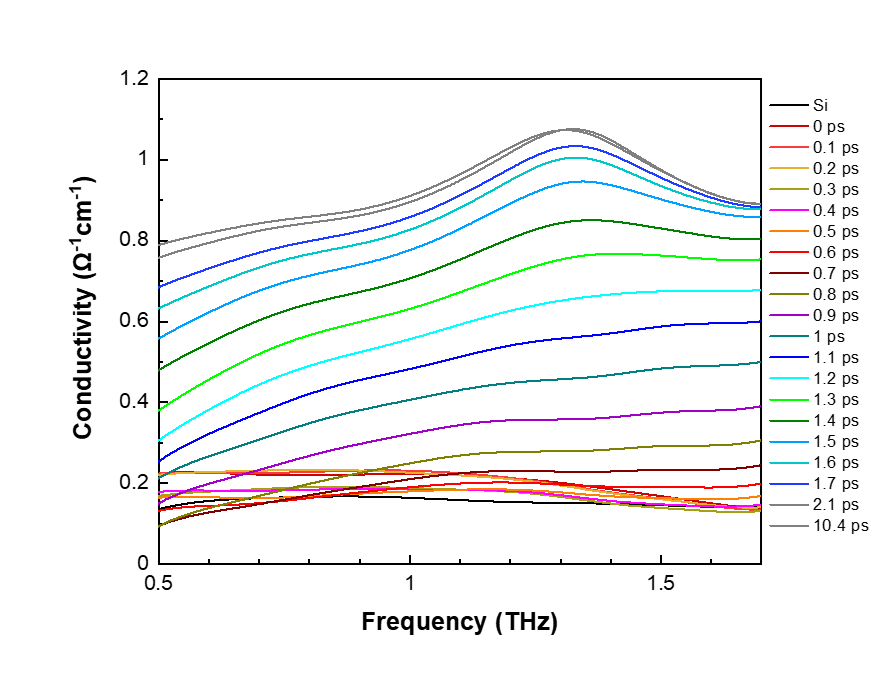
**Figure S1. Optical pump-induced photo conductivity of Si substrate.** Conductivity is calculated from dielectric measurement and Ohm’s law. $\varepsilon\left( \omega\right)=\varepsilon_{\infty}+\frac{i\sigma\left( \omega\right)}{\omega\varepsilon_{0}}$, $\sigma_{Re}\left( \omega\right)=\varepsilon_{o}\omega\varepsilon_{Im}\left( \omega\right)$.


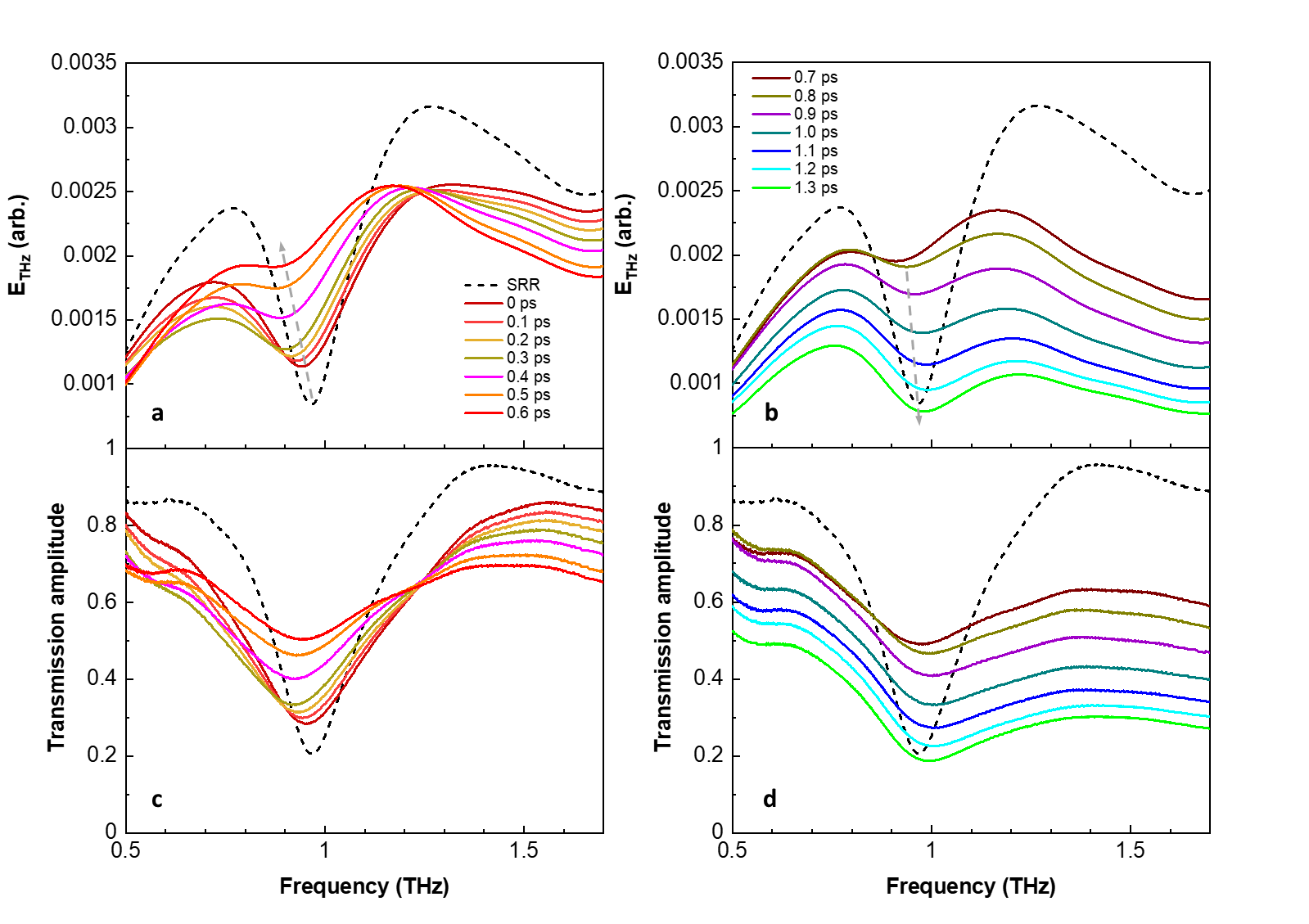


**Figure S2.** **Temporal changes in THz transmission of the SRR as a function of the pump-probe delay time.** (**a**) and (**b**) are transmitted THz spectra and (**c**) and (**d**) are transmission amplitude, which is defined as $t\left( \omega\right)=\frac{E_{SRR}\left( \omega\right)}{E_{Si}\left( \omega\right)}$, where $E_{SRR}\left( \omega\right)$ and $E_{Si}\left( \omega\right)$ are measured THz spectra of the sample and Si substrate, respectively. Spectral change in phase 1 (a, c) and phase 2 (b, d), respectively.


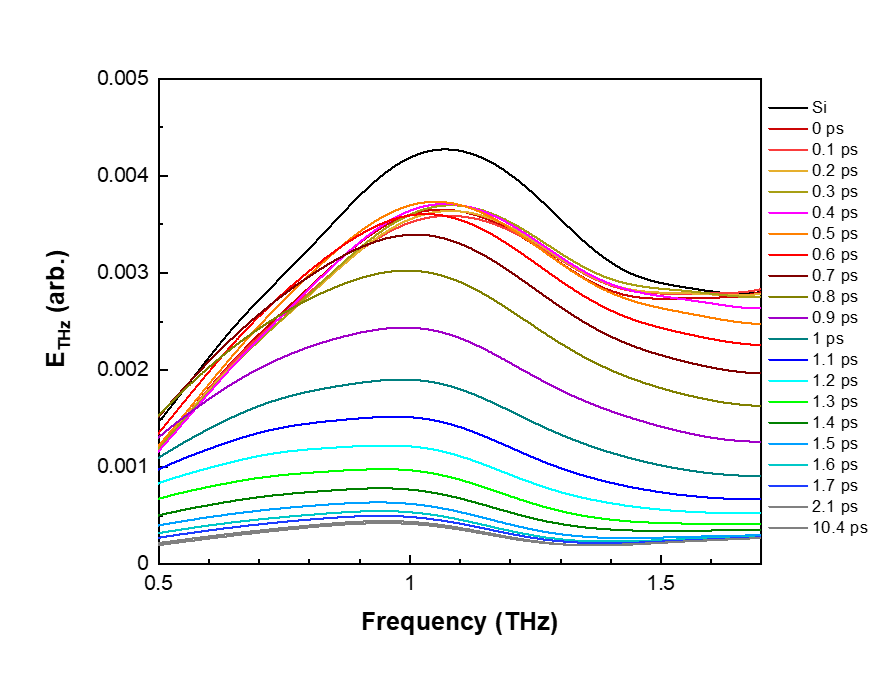
**Figure S3. THz field spectra transmitted through Si substrate for all delay times.**


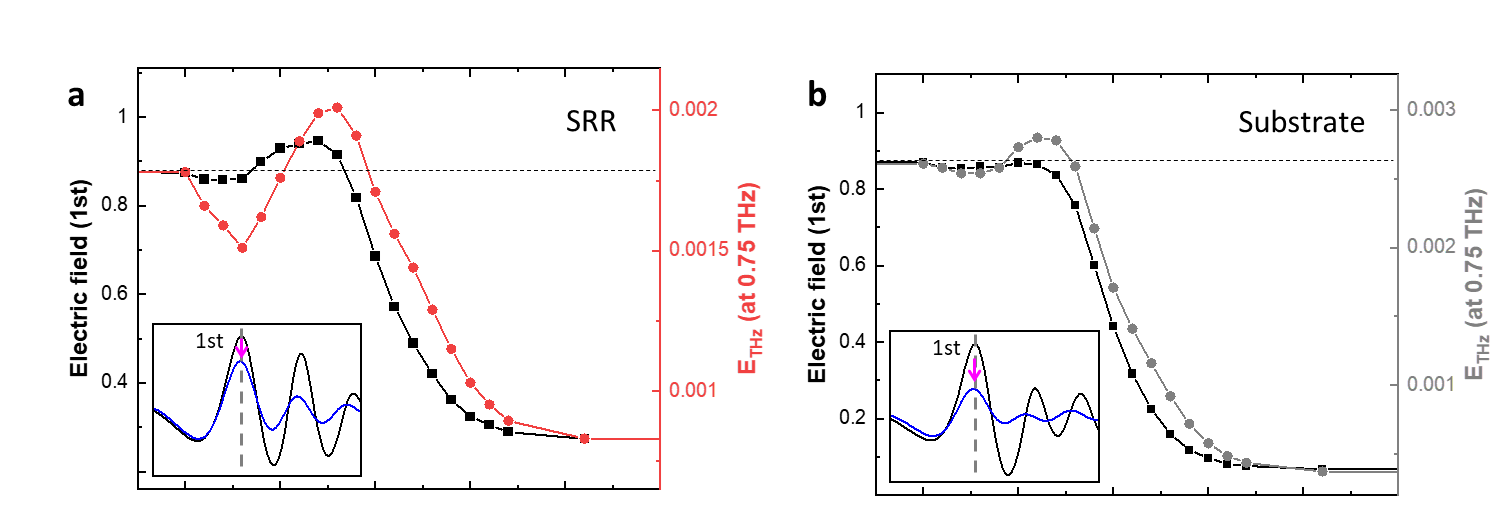


**Figure S4. Analysis of the time domain pulses. a,** A comparison is presented between the pump-induced change in the magnitude of the first peak in the time-domain pulse (represented by black scatter line, positions of peak is shown in the inset) with the corresponding spectral data (red scatter line) for the SRR case. The strong correlation between the two datasets indicates that the first peak of the THz pulse primarily consists of frequency components near 0.75 THz. The slight oscillating THz first peak change in the short pump–probe interval (phase 1 in Figure 2a) is attributed to the turnover behavior of the 0.75 THz component as the LC resonance gradually diminishes (Figure S2a, Supporting Information). **b,** The corresponding data for the Si substrate is represented.


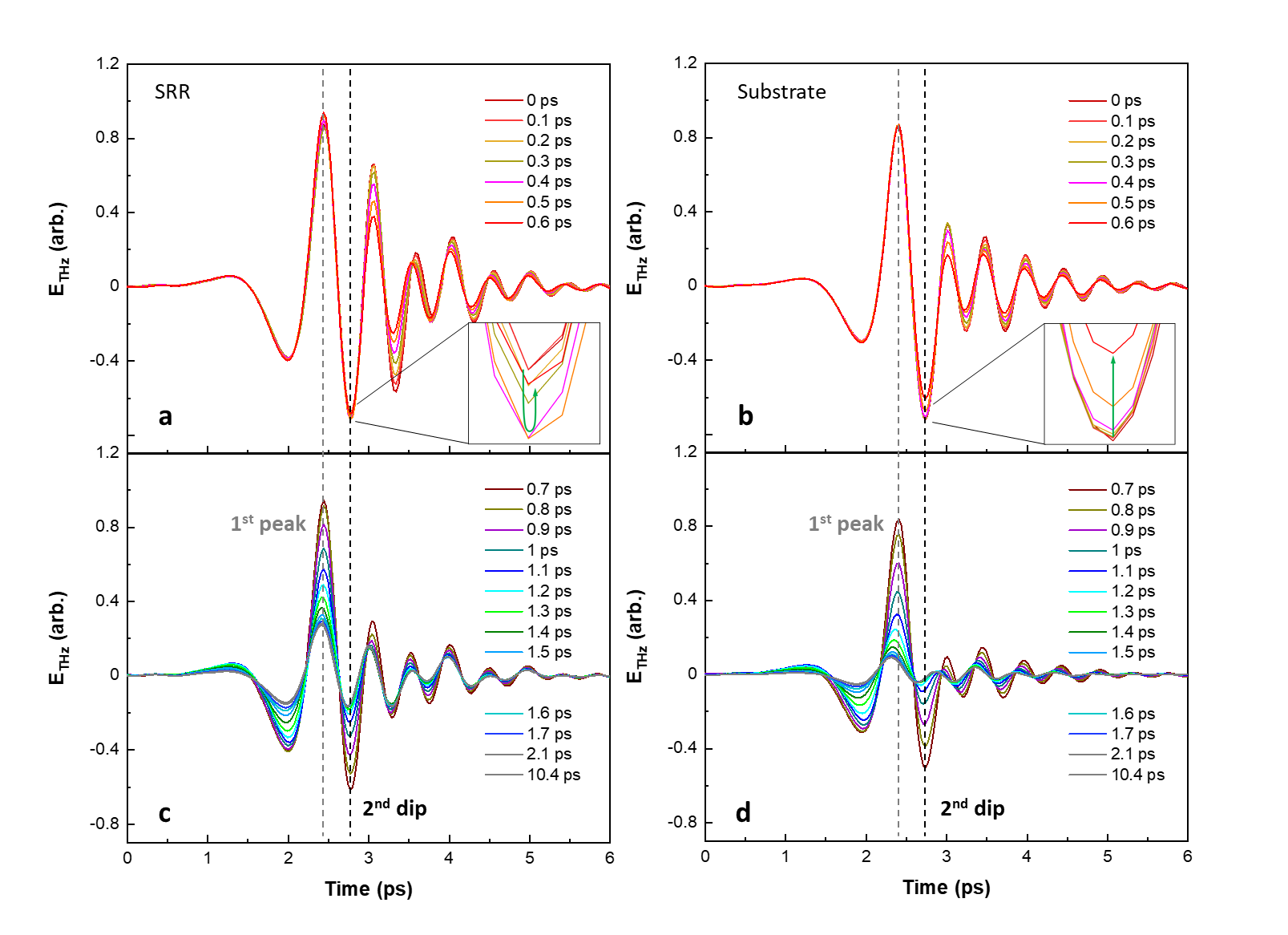
**Figure S5. Temporal changes in the time domain pulse.** THz pulse shapes transmitted through SRR within delay phase 1 (**a**) and phase 2-3 (**c**). Corresponding to the Si substrate within phase 1 (**b**) and phase 2-3 (**d**). Dashed lines denote location of 1^st^ peak and 2^nd^ dip, respectively. The colors of the spectral lines follow the arrow shown in Fig. 2a in the main text. The green arrow in the inset denotes temporal change of 2^nd^ pulse dip with pump delay times.


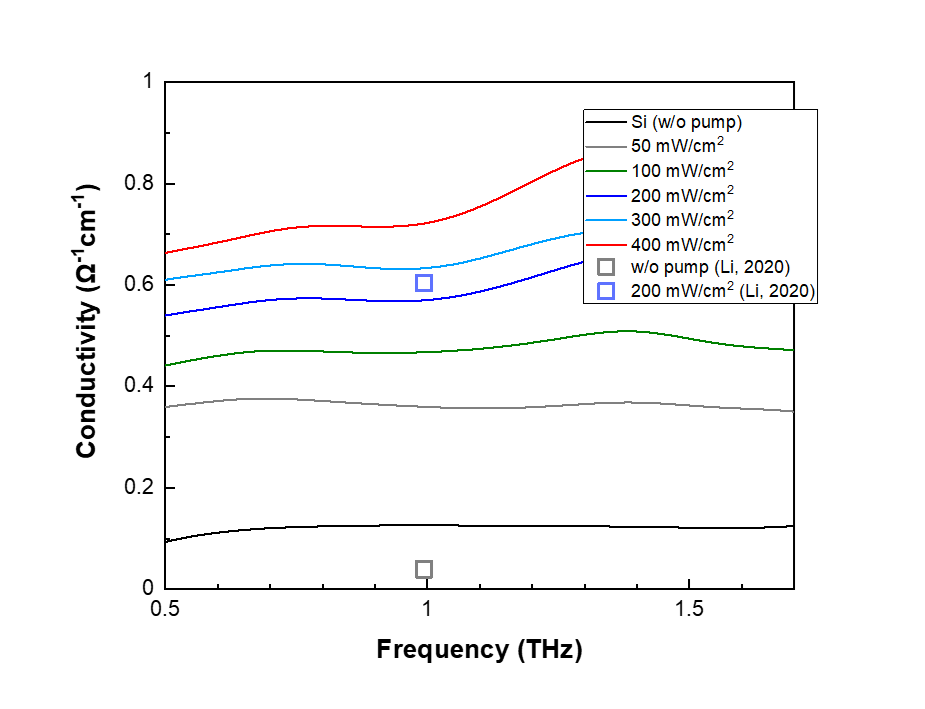


**Figure S6. Photon-induced conductivity of Si wafer as a function of the pump fluences.** Scatter is conductivity of high resistive Si wafer around 1 THz in previous study^[1]^.


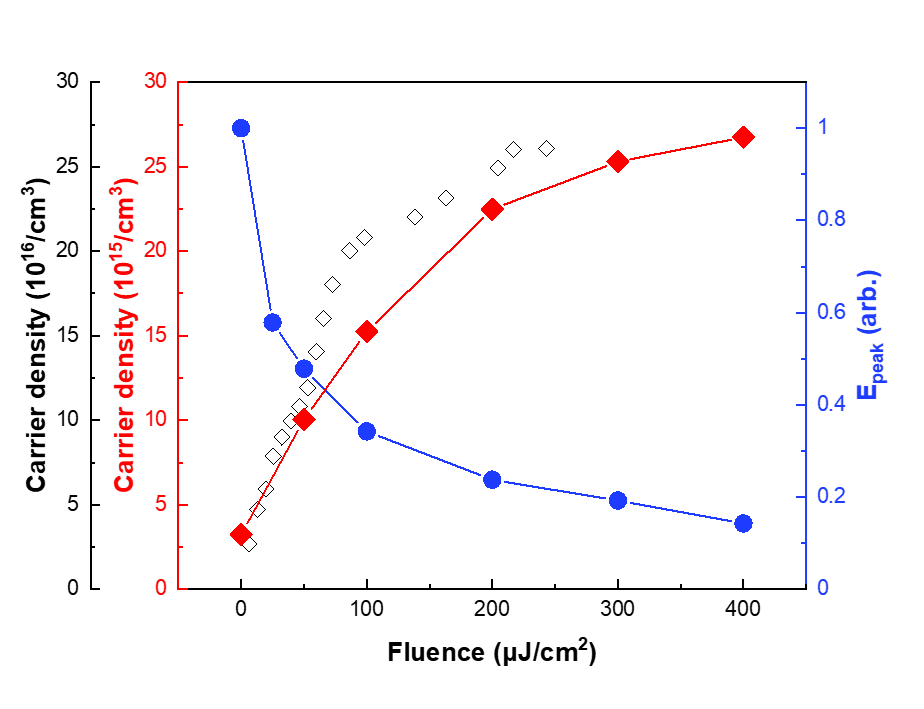


**Figure S7. Photon-induced carrier density (left) and THz field amplitude (right) of Si wafer as a function of the pump fluences, respectively.** Color-filled scatter represent the result of our high resistivity Si wafer (10 kΩ) and black-hollow scatter is data from previous study on 1 kΩ Si wafer^[2]^. All field amplitudes have been normalized to data without pumping. Carrier density was calculated from Drude model. Inverse correlation between carrier density and change of THz pulse peak is observed. Carrier density gradually saturate at higher pump fluences, which reproduces previous work. Please note that almost exactly 10 times less carrier density was observed in our Si wafer, whose resistivity is 10 times larger than in previous study.


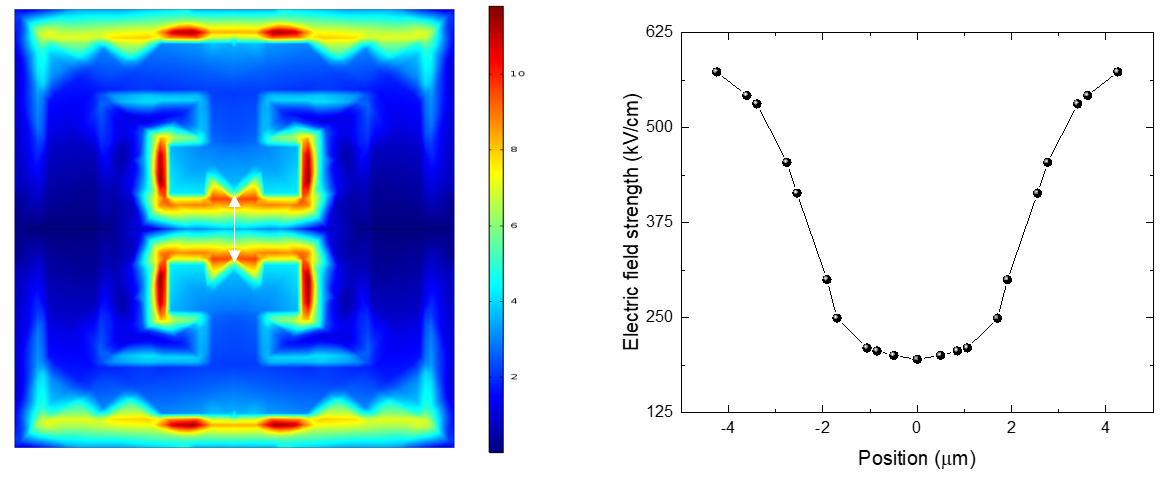


**Figure S8. THz field distribution at resonance frequency calculated by Finite-Difference Time-Domain (FDTD) method and electric field strength of position from the center of the gap (white arrow from left figure).**


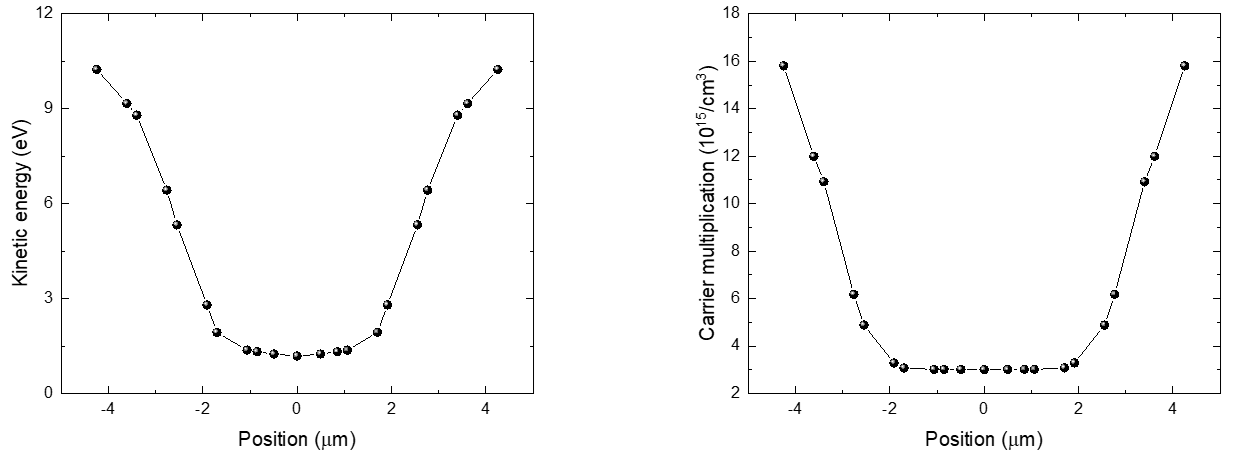


**Figure S9. Kinetic energy of electron in silicon and carrier concentration from THz field at distribution at position from the center of the gap.**

Impact ionization plays a critical role in devices like avalanche photodetectors, which rely on strong electric fields and high carrier drift velocities to generate electron-hole pairs. This process is the inverse of Auger recombination, as it utilizes the kinetic energy of another electron or hole to create additional electron-hole pairs. The impact ionization rate, 𝐺(𝐸), is typically expressed as^[3]^:

$$G\left( E \right)=\alpha_{n}\left( E \right)n\mu_{n}+\alpha_{p}\left( E \right)p\mu_{p}$$

where α_n_(E) and α_p_(E) are impact ionization coefficients for electrons and holes, E is electric field strength (V/cm), n and p are electron and hole concentrations, μ_n_​ and μ_p_​: electron and hole mobilities. The ionization coefficients (α_n_(E) and α_p_(E)) depend strongly on the electric field and total ionization coefficient can be approximated as:

$$a_{n,p}\left( E \right)=A\times exp[-\left( \frac{B}{E} \right)]$$

where A is coefficient representing the probability of ionization, B is effective energy threshold, respectively. For silicon, typical values of A is 3.8 x 10^6^ cm^-1^ and B is 1.75 x 10^6^ V/cm from ref. [3] The carrier concentration N(E,t) due to impact ionization is governed by:

$$\frac{dN\left( E,t \right)}{N\left( E,t \right)}=a_{n,p}\left( E \right)vdt=A\times exp[-\left( \frac{B}{E} \right)]vdt$$

where v is drift velocity of electron in silicon 1.05 × 10^7^ cm/s , t is duration of the applied THz field. The final carrier concentration, considering an initial concentration N(0) is given by

$$N\left( E,t \right)=N\left( 0 \right)\times\exp\left[ a_{n,p}\left( E \right)\cdot vt \right]=N\left( 0 \right)\times\exp\left[ A\times exp[-\left( \frac{B}{E} \right)]\cdot vt \right]$$

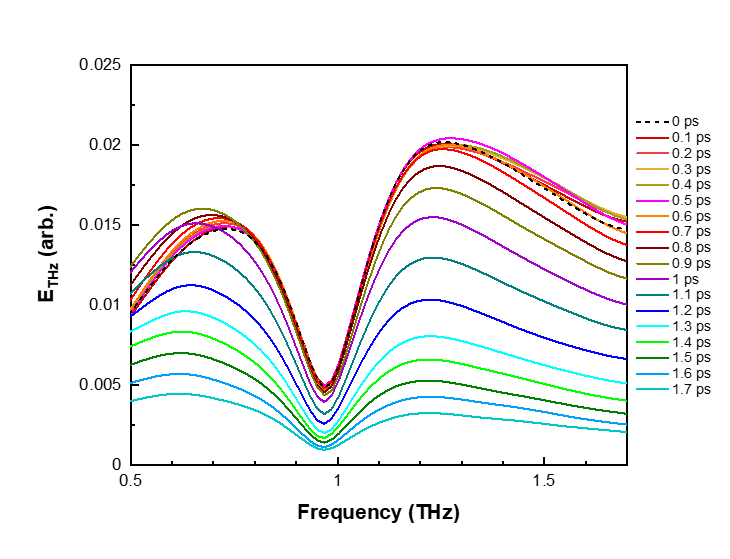


**Figure S10. THz amplitude modulation in case of back side optical pumping.** In contrast to optical pumping at the front side, no shorting out effect of the resonance is observed. Instead, a monotonic decrease in transmittance due to the metallization of the silicon substrate is observed.


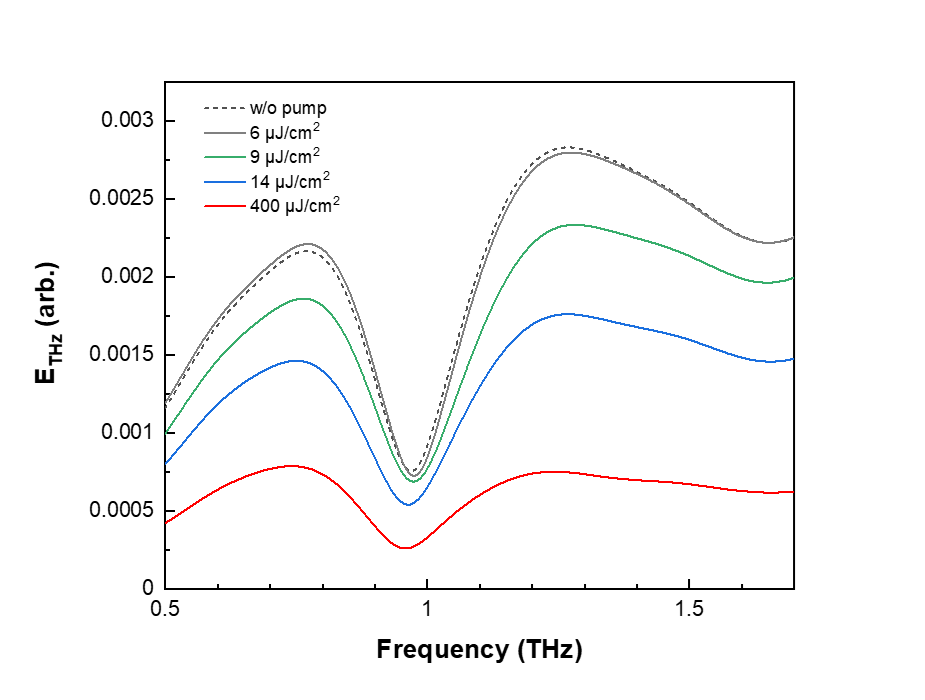


**Figure S11. Reduction of THz transmission of SRR measured at a pump-induced steady state (Δt = 10.4 ps, phase 3 in Fig. 2a) as a function of the pump fluences.**


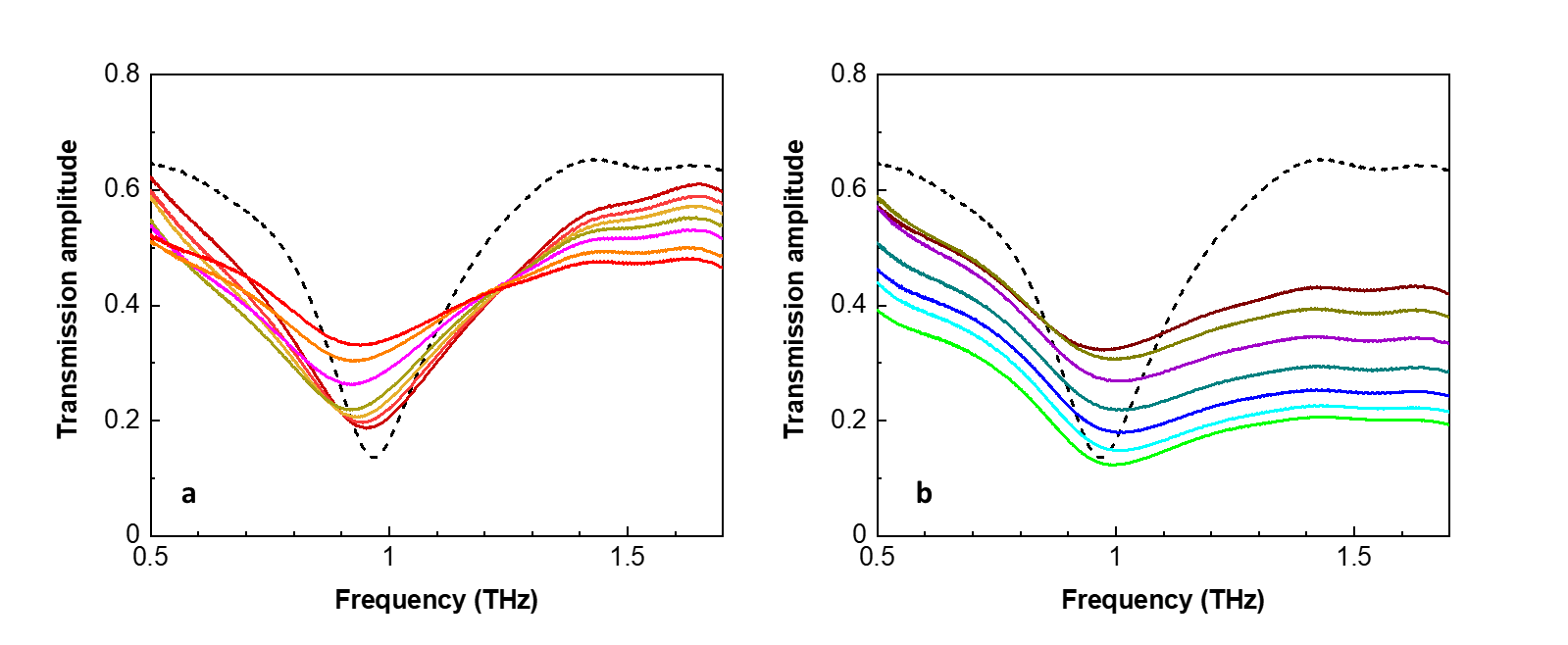


**Figure S12. Temporal changes in THz transmission of the SRR with respect to air.** (**a**) and (**b**) are transmission amplitude, which is defined as $t\left( \omega\right)=\frac{E_{SRR}\left( \omega\right)}{E_{Air}\left( \omega\right)}$, where $E_{Air}\left( \omega\right)$ is measured THz spectra of the dry air. Spectral change in phase 1 (a) and phase 2 (b), respectively.

**Supporting References**

[1] J. S. Li, M. S. Hu, *Sci. Rep.* **2020**, *10*, 6605.

[2] T. Moriyasu, M. Tani, H. Kitahara, T. Furuya, J. Afalla, T. Kohmoto, D. Koide, H. Sato, M. Kumakura, *Opt. Commun.* **2024**, *554*, 130139.

[3] S. M. Sze, K. K. Ng, *Physics of Semiconductor Devices*, Wiley-Interscience, Hoboken, NJ **2007**.
